# Optimization of the spherical integrity for sustained-release alginate microcarriers-encapsulated doxorubicin by the Taguchi method

**DOI:** 10.1038/s41598-020-78813-1

**Published:** 2020-12-10

**Authors:** C. T. Pan, S. T. Chien, T. C. Chiang, C. K. Yen, S. Y. Wang, Z. H. Wen, C. Y. Yu, Y. L. Shiue

**Affiliations:** 1 Department of Mechanical and Electro-Mechanical Engineering, National Sun Yat-Sen University, Kaohsiung, Taiwan; 2 Institute of Medical Science and Technology, National Sun Yat-Sen University, Kaohsiung, Taiwan; 3 Department of Pathology, Kaohsiung Armed Forces General Hospital, Kaohsiung, Taiwan; 4 Institute of Biomedical Sciences, National Sun Yat-Sen University, Kaohsiung, Taiwan; 5 Department of Marine Biotechnology and Resources, National Sun Yat-Sen University, Kaohsiung, Taiwan; 6 Liver Transplantation Program and Departments of Diagnostic Radiology and Surgery, Kaohsiung Chang Gung Memorial Hospital, and Chang Gung University College of Medicine, Kaohsiung, Taiwan; 7 Institute of Precision Medicine, National Sun Yat-Sen University, Kaohsiung, Taiwan

**Keywords:** Cell biology, Oncology, Engineering, Materials science

## Abstract

This study aimed to develop biodegradable calcium alginate microcarriers with uniform particle size and spherical integrity for sustained-release targeting transarterial chemoembolization. To determine related parameters including the ratio of cross-linking volume (sodium alginate: CaCl_2_), concentrations of sodium alginate and CaCl_2_ solutions, collection distance, flow rate, stirring speed, syringe needle diameter and hardening time to fabricate the microcarriers, the Taguchi method was applied. Using different conditions, a total of 18 groups were prepared. The average size of microspheres from different groups was estimated as ~ 2 mm (range 1.1 to 1.6 mm). Signal-to-noise ratio analysis showed the optimal spherical integrity (F1) achieved when the above parameters were designed as 0.1, 2.5 wt%, 6 wt%, 8 cm, 30 mL/h, 150 rpm, 0.25 mm and 2 h, respectively. The best (F1), middle (F2) and worst (F3) groups were used for further experiments. Fourier-transform infrared spectroscopy spectrum showed that F1, F2 and F3 conformations were distinct from original sodium alginate. Drug-loaded calcium alginate microcarriers demonstrated rougher surfaces compared to microspheres without drug under transmission electron microscopy. Compared to pH 7.4, swelling rates in PBS were decreased at pH 6.5. Encapsulation and loaded efficiencies of the Dox-loaded microcarriers were estimated as ~ 40.617% and ~ 3.517%. In vitro experiments indicated that the F1 Dox-loaded microcarriers provide a well sustained-release efficacy for about two weeks at 37 °C in PBS. Treatments of calcium alginate microcarriers without the Dox in two distinct hepatocellular carcinoma-derived cell lines, Huh-7 and Hep-3B, indicated that these microcarriers were non-toxic. The Dox-loaded microcarriers displayed sustained-release capacity and reduced cell viabilities to ~ 30% in both cell lines on Day 12.

## Introduction

Hepatocellular carcinoma (HCC) is one of the most common cancers in the world, which is usually diagnosed at its advanced stages. Transarterial chemoembolization (TACE) is a commonly used, minimally invasive treatment for HCC patients^1^. TACE can deliver microcarriers loaded with chemotherapeutic drugs including doxorubicin (Dox), cisplatin or mitomycin into the hepatic artery to form an embolism and induce hypoxia, blocking off the blood and nutrient supplies to the tumor^2^. Chemoembolisation especially using Dox, improved survival of stringently selected patients with unresectable hepatocellular carcinoma^3^.

Modern embolic agents are either temporary or permanent. Commercial drug-loaded microspheres such as the HepaSphere™ and the DC BEAD™ for TACE in HCCs, which were approved by the Food and Drug Administration (FDA, USA)^4,5^, are expensive and non-biodegradable. Therefore, fabricating biodegradable microspheres to prevent permanent embolization, compression of normal organs and arteries by drug-loaded microspheres and postoperative complications are extremely important. Various types of biocompatible and biodegradable polymers such as chitosan^6^, gelatin^7^, chitooligosaccharide^8^ and sodium alginate^9^ have been used to prepare microcarriers for drug delivery systems. Alginates are classified ‘Generally Recognized as Safe’ in food processing, which is formed by a (1–4)-linked β-d-mannuronate (M) and C-5 epimer α-l-guluronate (G), a polymer containing M-, G- and MG-blocks. These blocks are composed of consecutive G residues, M residues and alternating G and M residues (GMGMGM)^10^. Being a non-toxic polyanionic polysaccharide, alginate shows to be biocompatible and highly hydrophilic, which is generally used as a stabilizer, viscosifier and gelling agent in food, textile, pharmaceutical and biotechnological industries^11^. Zhang et al. further confirmed that phenylalanine ethyl ester-coated alginate nanoparticles weren’t able to inhibit the proliferation of colon cancer cells even at a high concentration of 1000 μg/mL^12^. Calcium alginate gel is a biocompatible and stable polymer for endovascular embolization^13^. Due to these properties, sodium alginate has been used as a microcarrier for different biological agents, such as peptides^14^ and antigens^15^.

The water-based ion crosslinking technology provides characteristic advantages compared to conventional methods to prepare microspheres. It has been used for the preparation of alginate microspheres containing pindolol^16^ and genipin^17^. The Dox-loaded alginate microspheres showed a property of delayed-release of drug in the liver, prolonging the retention time and concentration of the Dox after embolization in vivo^18^. However, a high concentration of sodium alginate tended to form a macro-sized hydrogel^19^. Determining factors such as cross-linking volume ratio (sodium alginate: CaCl_2_), concentrations of sodium alginate and CaCl_2_ solutions, collection distance, flow rate, stirring speed, syringe needle diameter and hardening time and their interactions are critical for the successful preparation of alginate microspheres with high spherical integrity to render sustained-release capacities. Therefore, we aimed to identify the optimal parameters to fabricate such calcium alginate microcarriers.

## Results and discussion

### Optimization of spherical integrity by the Taguchi method

**Figure 1:**
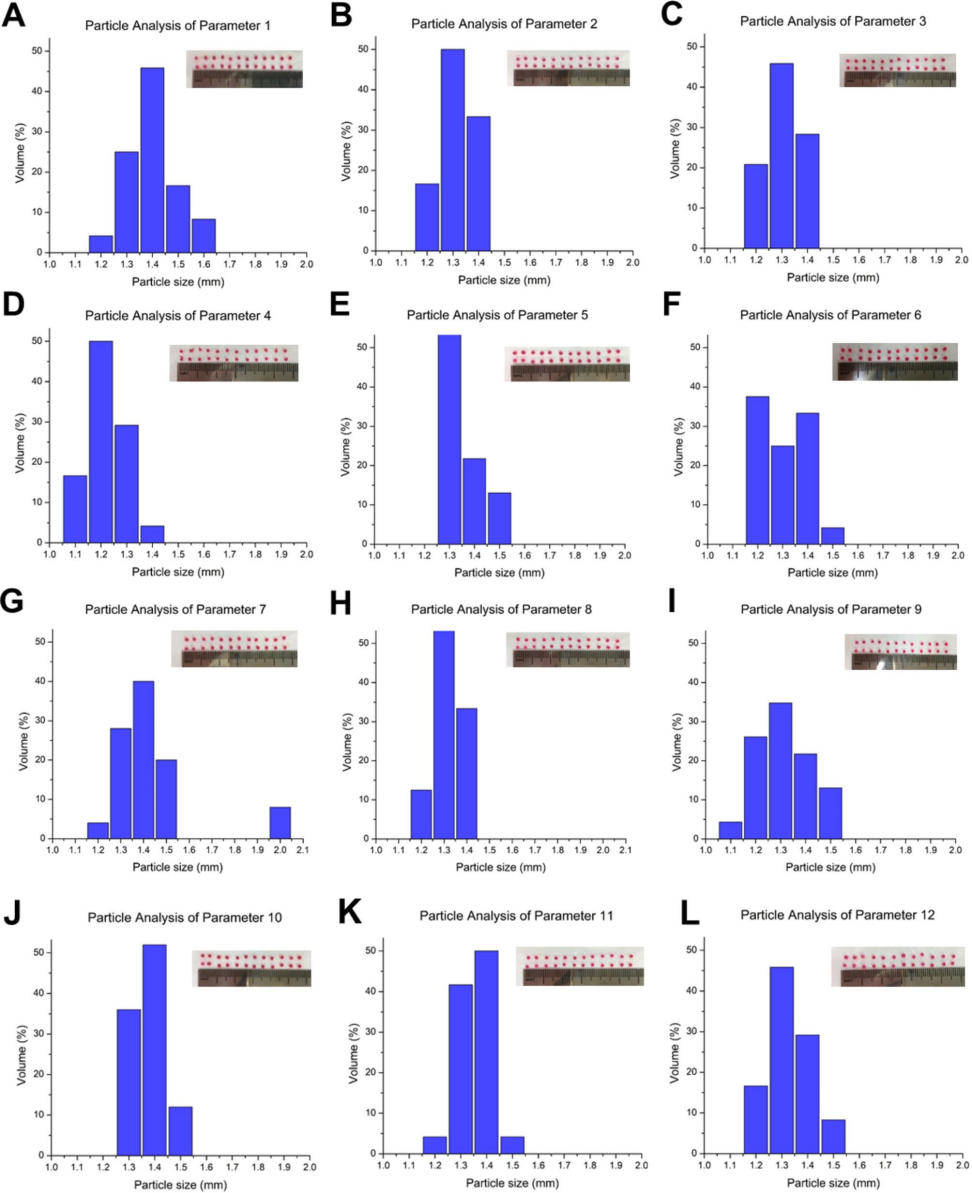

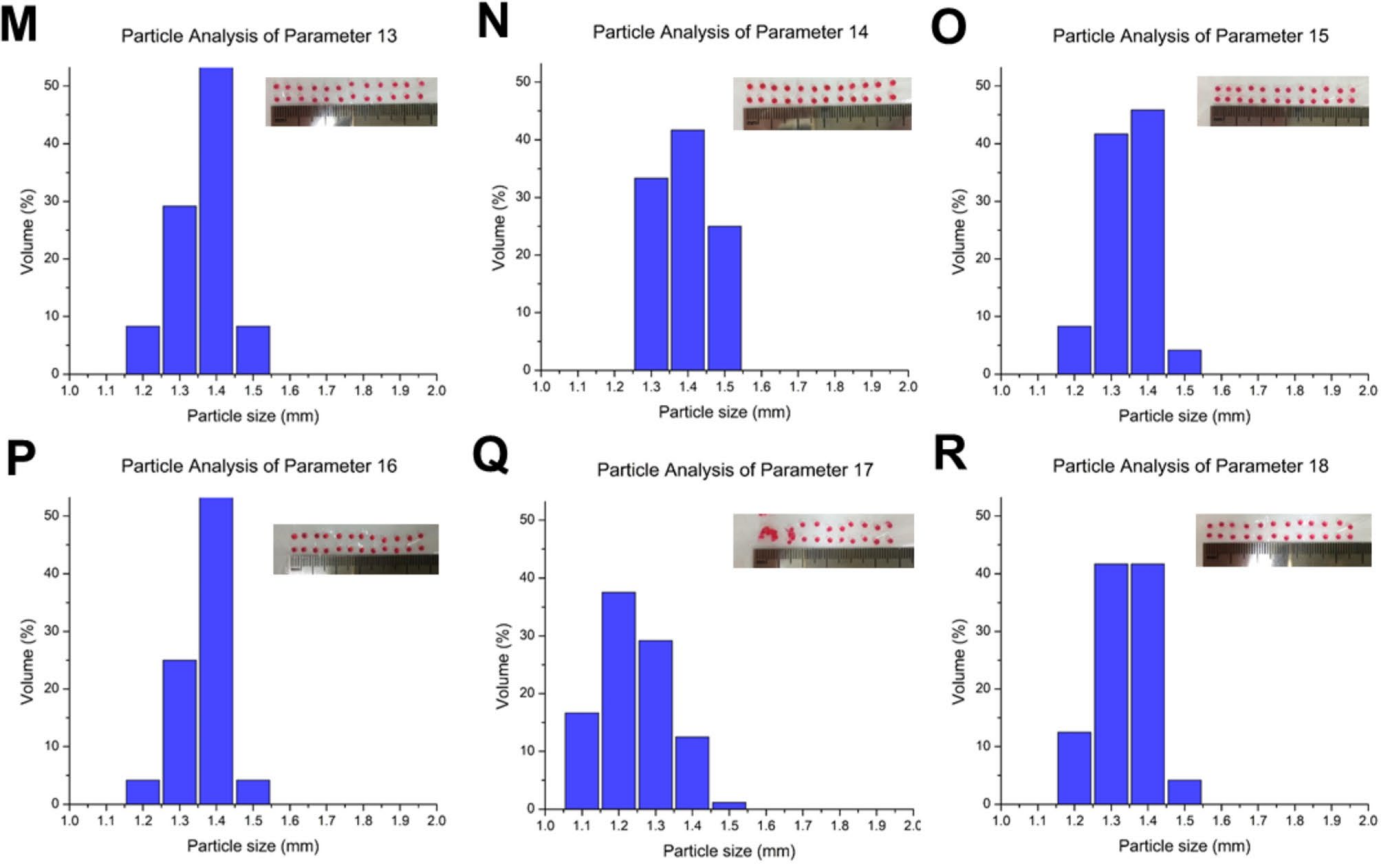
(**A**–**R**) The distributions of diameter (mm) and volume (%), and appearances of calcium alginate microcarriers with 18 different parameter combinations.

Calcium alginate microcarriers were prepared by the Taguchi method (Supplementary Table S1). The definition and trial levels of factors in Taguchi’s L_18_ orthogonal array experiment is shown in Table 1. A total of 18 sets of microcarriers with a particle size ~ 2 mm (range 1.1 to 1.6 mm) were prepared and are shown in Fig. 1A–R. Roughly, three types of microspheres were found: irregular (Fig. 1D,Q), nearly oval (Fig. 1G,H,M,N) and tear-drop (Fig. 1I,K). Microcarriers were next immersed in PBS for 14 days for further examination of their surface completeness. The definition of scoring criteria for the microsphere surface is listed in Table 2. A high score indicates a complete spherical shape after PBS immersion. All experiments were performed in triplicate and appearance scores were averaged and converted to signal-to-noise (*S/N*) ratios (Supplementary Table S2).

**Table 1:** Definition and trial levels of factors in Taguchi’s L_18_ orthogonal array experiment to fabricate calcium alginate microcarriers.

Code	Parameter	Level	Unit
A	Cross-linking volume ratio (Sodium alginate: CaCl_2_)	0.17, 0.1	–
B	Sodium alginate solution concentration	1.5, 2, 2.5	wt%
C	CaCl_2_ solution concentration	3, 6, 9	wt%
D	Collection distance	2, 5, 8	cm
E	Flow rate	30, 40, 50	mL/h
F	Stirring speed	100, 150, 200	rpm
G	Syringe needle diameter	0.25, 0.2, 0.15	mm
H	Hardening time	1, 2, 3	h

**Table 2:** Definition of scoring criteria for the microsphere appearance.

Score	Definition
1	The microcarriers dissolved and disappeared
2	More than half the microcarriers broke and became translucent
3	Less than half the microcarriers broke or became translucent
4	The microcarriers stayed intact and became translucent
5	The microcarriers stayed intact

Microcarriers from experiment #1 (Fig. 1A) and #17 (Fig. 1Q) became translucent after PBS immersion. Those from experiment #5, #6 and #14 (Fig. 1E,G,N) showed intact surfaces, indicating that they are structurally stable. We next converted the *S/N* ratios into a factor response table (Table 3) and found that the most critical factors regarding the surface integrity of microcarriers are the collection distance, hardening time, and the concentration of the CaCl_2_ solution. The factor response graph showed the *S/N* ratio for each parameter (Supplementary Fig. S1A). The contribution ratio of the D (collection distance) was 48.839%, followed by the H (hardening time): 16.395% and the C (CaCl_2_ solution concentration): 11.416% (Supplementary Fig. S1B), suggesting that these factors greatly impacted on scoring results of calcium alginate microcarriers. The analysis of the variance was performed based on the *S/N* and expressed as the contribution ratio (ρ%) (Supplementary Table S3). The definition of contribution ratio is the change of the relationship between factor effect and overall change. Different factors have different effects on quality characteristics, so the contribution ratio can be calculated to evaluate the effect of each factor. The combination of the optimal, middle and worst parameters were denoted by the F1, F2 and F3, as showed in Supplementary Table S4. The optimal parameters were achieved when the cross-linking volume ratio (sodium alginate: CaCl_2_), sodium alginate solution, CaCl_2_ solution, collection distance, flow rate, stirring speed, syringe needle diameter, hardening time was selected as 0.1, 2.5 wt%, 6 wt%, 8 cm, 30 mL/h, 150 rpm, 0.25 mm and 2 h, respectively (Supplementary Table S4). Accordingly, F3 showed the worst surface integrity.

**Table 3:** Factor response table.

	A	B	C	D	E	F	G	H
Level 1	11.297	10.862	10.603	9.442	11.832	10.506	12.017	11.093
Level 2	11.372	11.517	12.177	12.197	11.134	11.873	10.777	12.382
Level 3		11.624	11.223	12.363	11.037	11.624	11.208	10.528
Effect	0.075	0.762	1.575	2.921	0.795	1.368	1.240	1.855
Rank	8	7	3	1	6	4	5	2

A. Cross-linking volume ratio (Sodium alginate: CaCl_2_), B. Sodium alginate solution concentration, C. CaCl_2_ solution concentration, D. Collection distance, E. Flow rate, F. Stirring speed, G. Syringe needle diameter and H. Hardening time.

### The structures of calcium alginate microcarriers and low environmental pH value notably decrease the swelling rates

**Figure 2:**
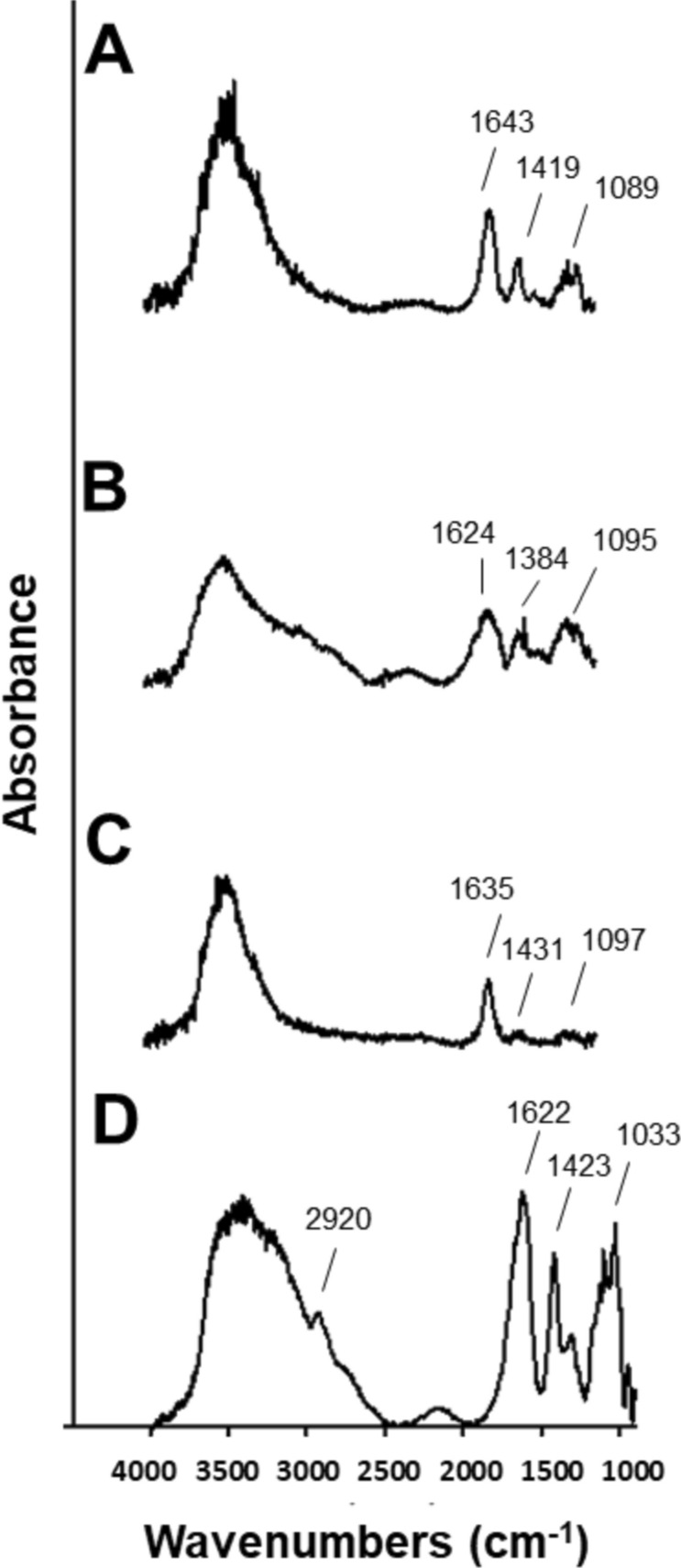
Fourier-transfrom infrared spectroscopy (FTIR) spectra of calcium alginate microcarriers fabricated with different parameters (**A**) F1, (**B**) F2 and (**C**) F3, indicates that F1, F2 and F3 contain the –OH (3000 to 3600 cm^−1^), –COO– (1342, 1423, 1622 cm^-1^), –C–C–H, –C–O–H, –C–O (935 to 1107 cm^−1^) linkages compared to sodium alginate (**D**), and F1 (**A**) is more similar to control (**D**).

Three types of calcium alginate microcarriers were prepared by using the parameters F1, F2 and F3; uneven and porous surfaces were observed in all groups (Fig. 1A–R). Subsequently, microcarriers were collected after drying at 55 °C. Alterations of the chemical structure of sodium alginate and calcium alginate microcarriers after cross-linking with calcium chloride were studied by Fourier-transform infrared spectroscopy (FTIR) spectrum (Fig. 2). An absorption of 3000–3600 cm^−1^ indicates the tensile vibration of the hydroxyl group (–OH) in the sodium alginate structure. In the absorption sections of 1342 cm^−1^, 1423 cm^−1^ and 1622 cm^−1^, asymmetric and symmetric tensile vibrations with the carboxylate ion (–COO–) structure were observed. The absorption section at 935–1107 cm^−1^ represents the deformation of the –C–C–H and –C–O–H of the pyranosyl ring and the stretching vibration of –C–O in the sodium alginate structure, and this absorption wavelength can be used to distinguish the structure of alginic acid. Figure 2A–C show that the absorption wavelength regions of the hydroxyl stretching vibration of the microcarriers were narrower than that of sodium alginate (Fig. 2D). Because the hydroxyl and carboxylate groups of sodium alginate were chelated by calcium ions, the tensile vibration of -OH was reduced. The results of FTIR demonstrated that the formation of calcium alginate microcarriers was ionic cross-linked and the chemical bonding remained. Although it has been well known that the alginate macromolecules cross-links with calcium cations when they are in-contact, in this study, we focused on the effect of different combinations of the parameters (F1, F2 and F3) on absorbance profiling. Undeniably, calcium alginate microcarriers are hydrogels and a previous study indicated that hydrogel materials showed poor mechanical properties compared to those of solid polymers [reviewed in^20^].

**Figure 3:**
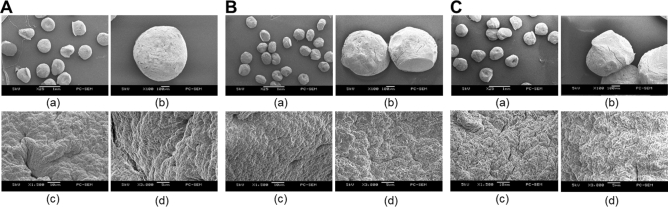
Scanning electron microscope (SEM) images of Dox-loaded calcium alginate microcarriers using parameter of **(A)** F1, **(B)** F2 and **(C)** F3, respectively, and F3 shows dense wrinkles thus increased surface area compared to F1 and F2.

Scanning electron microscope (SEM) identified that the F1 Dox-loaded calcium alginate microcarriers were shrunk due to the drying process and became irregular. Cracks were also found on the surface (Fig. 3A). These results could be anticipated and were consistent with another study, especially for microcarriers prepared with low alginate concentrations^21^. The drying process affected many properties such as size, shape, mechanical character, swelling state and drug-releasing of the microcarriers. However, much smoother surfaces were found in the calcium alginate microcarriers without the Dox (Supplementary Fig. S2) compared to those with the Dox (Fig. 3). High surface roughness in the F2 and F3 Dox-loaded calcium alginate microcarriers are shown in Fig. 3B,C. Although the microsphere surfaces observed by SEM may be more uneven compared to the original porous surfaces, it is worth to compare among different parameter combinations. Drug-loaded calcium alginate microcarriers showed rougher surfaces compared to microspheres without drug, consistent with those were observed in indomethacin-calcium alginate microdiscs^22^ and ceftriaxone sodium-loaded calcium alginate beads^23^. Among the three groups, the surface of Dox-loaded F3 calcium alginate microcarriers was the roughest due to the low concentration of sodium alginate solution used in the process of preparation. As the surface wrinkles of the microcarriers increased, the surface area also expanded, resulting in their high exposure to external environment. These observations suggested that the surface area of the calcium Dox-loaded alginate microcarriers may affect the drug release rate and their biodegradation.

**Figure 4:**
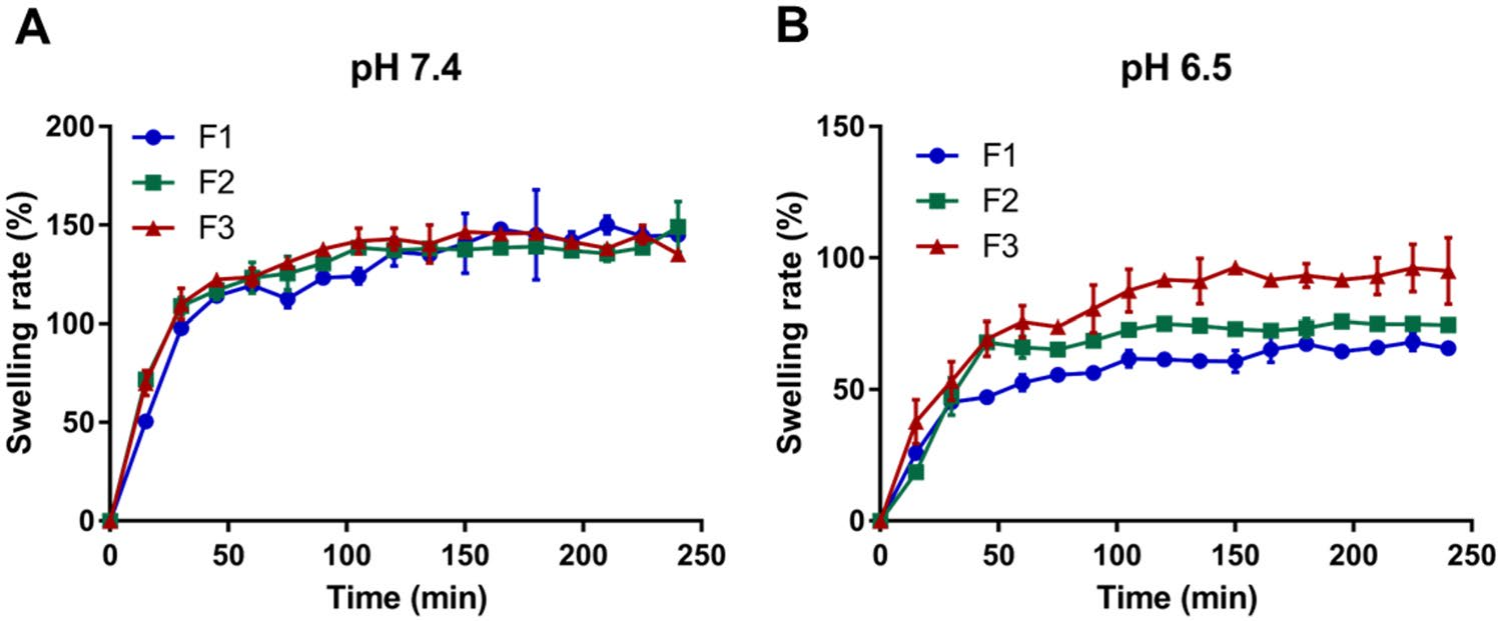
Low pH value notably decreases the swelling rates in PBS at 37 °C. The swelling rates of calcium alginate microcarriers after PBS immersion from 0 to 240 min were measured. (**A**) In pH 7.4 PBS, the maximal swelling rates were reached at 240 min for F1 (145.13% ± 1.237) and F2 (149.13% ± 12.904) while at 180 min for F3 (146.13% ± 1.590). (**B**) In pH 6.5 PBS, the maximal swelling rates were reached at 240 min for F1 (65.75% ± 2.828), F2 (73.44% ± 0.353) and F3 (95.00% ± 12.72), respectively.

Recent studies suggested that the tumor formation is accompanied by an increase of glucose uptake and lactate accumulation, resulting in low pH in the microenvironment, called the ‘Warburg effect’^24^. Therefore, we prepared two PBS solutions with pH 7.4 (normal body fluids) and pH 6.5 (tumor microenvironment), respectively, to measure the swelling rates of microcarriers at hydrogel state. The swelling rates of the F1, F2 and F3 calcium alginate microcarriers in PBS solution with pH 7.4 and pH 6.5 were measured in Fig. 4 from 0 to 240 min after immersion. As shown in Fig. 4A, microcarriers rapidly expanded within 30 min in pH 7.4 PBS, the swelling rate of the F1, F2 and F3 calcium alginate microcarrier were estimated as 97.88% ± 3.01, 108.88% ± 4.42 and 110.13% ± 7.95, respectively. During 45 min to 90 min after immersion, the F1, F2 and F3 calcium alginate microcarriers were continually expanding, but the swelling rates were gradually slowing down. After 105 min, the swelling rates of the microcarriers were almost steady in all groups. The maximal swelling rates of F1 and F2 calcium alginate microcarriers at 240 min were 145.13% ± 1.24 and 149.13% ± 12.90. However, the microcarriers began to disintegrate due to overexpansion and the swelling rate/relative weight of the F3 calcium alginate microcarrier was reduced by 10.88% at 240 min compared to that at 180 min.

The swelling rates of the calcium alginate microcarriers measured in PBS with pH 6.5 are shown in Fig. 4B. Similarly, the microcarriers rapidly expanded within 30 min and the swelling rates at 30 min of the F1, F2 and F3 calcium alginate microcarriers were 45.25% ± 1.06, 47.38% ± 7.25 and 53.25% ± 7.42, respectively. During 45 to 60 min, the swelling rates of the F1, F2 and F3 calcium alginate microcarriers slowly increased. The maximal swelling rates of the F1, F2 and F3 calcium alginate microcarriers at 240 min were 65.75% ± 2.83, 73.44% ± 0.35 and 95.00% ± 12.72, respectively. Thus in PBS with pH 7.4, the microcarriers absorb PBS rapidly, and the swelling rate was greater than 100% at 240 min after immersion. On the other hand, the swelling rates were fewer than 100% in PBS with pH 6.5. The calcium alginate microcarriers were pH-sensitive and low pH value notably decreases the swelling rates. Accordingly, it is suitable to serve as a microcarrier for drug delivery systems in cancer therapy. Indeed, alginate/poly(γ-glutamic acid) composite microparticles could absorb hundreds of times their weight in water. Both the maximum water up-take ratio and the swelling rate were increased with the increase of the poly(γ-glutamic acid) amount in the composite^25^. By immersing the microsphere (quercetin/chitosan/sodium alginate) in three respective PBS, simulated gastric fluid pH 1.2 and simulated intestinal fluid pH 6.8 and pH 7.4, the swelling index was decreased as the pH value decreased in three different groups with distinctive formulations^26,27^, consisting with our observations. This aspect may be explained by the acid environment protonated the carboxylate groups of the polymer localized on the surface of microspheres. The insolubility of alginic acid in the fluid and the formation of hydrogen bonds, may increase the structure stability, impede the penetration of additional fluid into the deeper layer of microspheres^27^.

### The Dox encapsulation and loaded efficiencies are highly correlated and fetal bovine serum in PBS markedly enhances drug release of the Dox-loaded calcium alginate microcarriers in vitro

**Figure 5:**
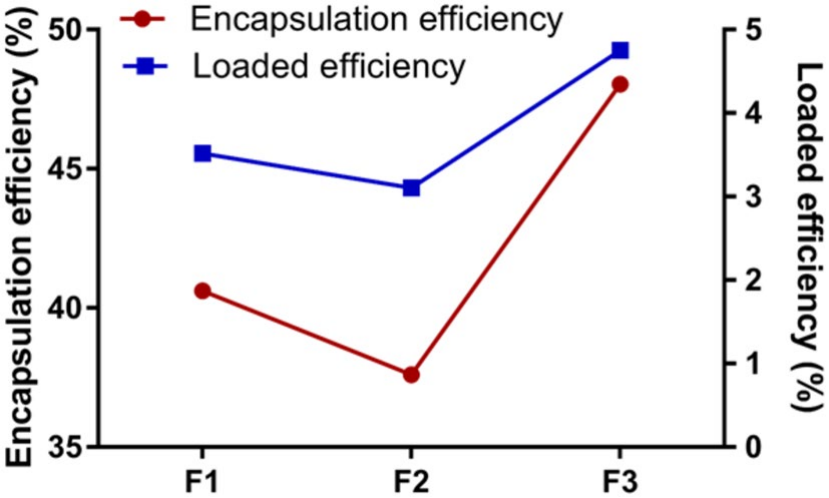
Encapsulation efficiencies are highly correlated to loading efficiencies of Dox with parameter of F1, F2 and F3, respectively. Microcarriers produced with F3 parameter shows the highest encapsulation efficiencies and loading efficiencies. Both efficiencies are highly correlated to each other in all groups.

Both drug encapsulation and loaded efficiencies are critical indexes for drug delivery systems, especially for expensive drugs. The encapsulation and loaded efficiencies of the F1, F2 and F3 Dox-loaded calcium alginate microcarriers are shown in Fig. 5 and Supplementary Table S5. As shown in Fig. 5, the F3 Dox-loaded calcium alginate microcarriers showed the highest encapsulation efficiency (up to ~ 48.05% ± 0.76) compared to the F1 (~ 40.62% ± 0.85) and the F2 (~ 37.61% ± 0.71) Dox-loaded calcium alginate microcarriers. The loaded efficiency of the F1, F2 and F3 were 3.52% ± 0.13, 3.11% ± 0.14 and 4.73% ± 0.25, respectively. Encapsulation and loaded efficiencies are highly correlated with each other in all groups (Fig. 5).

**Figure 6:**
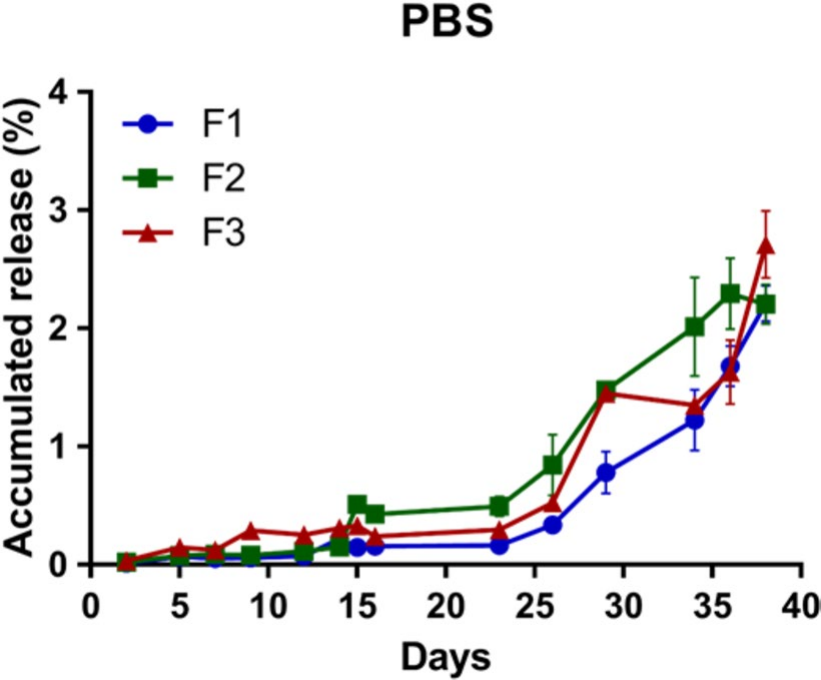
Dox release was slow before day 20 in PBS in F1, F2 and F3, surged after day 23.

In vitro, the Dox release from the calcium alginate microcarriers was carried out in the 1.5 mL of PBS (pH 7.4) solution and the PBS solution containing 10% FBS at 37℃ for one month. As shown in Fig. 6, the F1, F2 and F3 calcium alginate microcarriers were slowly released in PBS during the first two weeks. The cumulative release rates of the F1, F2 and F3 calcium alginate microcarriers were estimated as 0.15% ± 0.06, 0.51% ± 0.00 and 0.33% ± 0.10 at the end of the second week, respectively. At this time point, the drug release rate of the F2 calcium alginate microcarriers was the highest, probably because the hardening time of the F2 calcium alginate microcarriers was the shortest during the process. After 25 days, burst releases were observed in the F2 and F3 calcium alginate microcarriers, whereas the drug release rate of the F1 calcium alginate microcarriers remained slow. The cumulative release rates of the F1, F2 and F3 calcium alginate microcarriers on day 38 were 2.21% ± 0.02, 2.21% ± 0.01 and 2.71% ± 0.06, respectively. The F1 and F2 released the Dox gradually from day 12 to 38, while the F3 released the Dox abruptly during day 26 to 30 and day 35 to 40 (probably due to its rough surface), indicating that the F1 and F2 were more suitable as carriers for sustained drug release compared to the F3 calcium alginate microcarriers even though the F3 showed good encapsulation and loaded efficiencies.

The aim of adding 10% FBS to PBS was to simulate an environment of cell culture. The drug release curve was quite slow before day 12, and the cumulative drug release rates of the F1, F2 and F3 were 0.68% ± 0.40, 0.94% ± 0.22 and 1.26% ± 0.00, respectively. In particular, burst releases after day 12 was observed in all groups. On day 14, the cumulative drug release rates of the F1, F2 and F3 calcium alginate microcarriers were 10.35% ± 4.35, 19.33% ± 3.02 and 14.20% ± 6.08, respectively, much higher than those in PBS without FBS (data not shown). Perhaps due to FBS contains lipoproteins or other proteins/enzymes and destabilized the structure of calcium alginate microcarriers, leading to faster drug release. Also, PBS containing FBS enhanced PBS contamination with unknown microorganisms, resulting in large error bars starting day 14 and the experiment had to be terminated on day 16. The difference among all groups became meaningless. Therefore, to avoid contamination in the medium containing FBS is extremely critical. The releasing rate before Dima et al. studies showed that when the microcarriers were immersed in the solution, the internal and external osmotic pressure could be affected by the properties of the encapsulating material and the conditions (pH, ionic strength, temperature and surrounding environments)^28^, consistent with our observations. Therefore, the characteristics of swelling and releasing confirmed that these microcarriers are porous. Next, the F1 and F3 calcium alginate microcarriers were selected for in vitro cell experiments for further comparison.

### Calcium alginate microcarriers inhibit cancer cell viabilities in vitro

**Figure 7:**
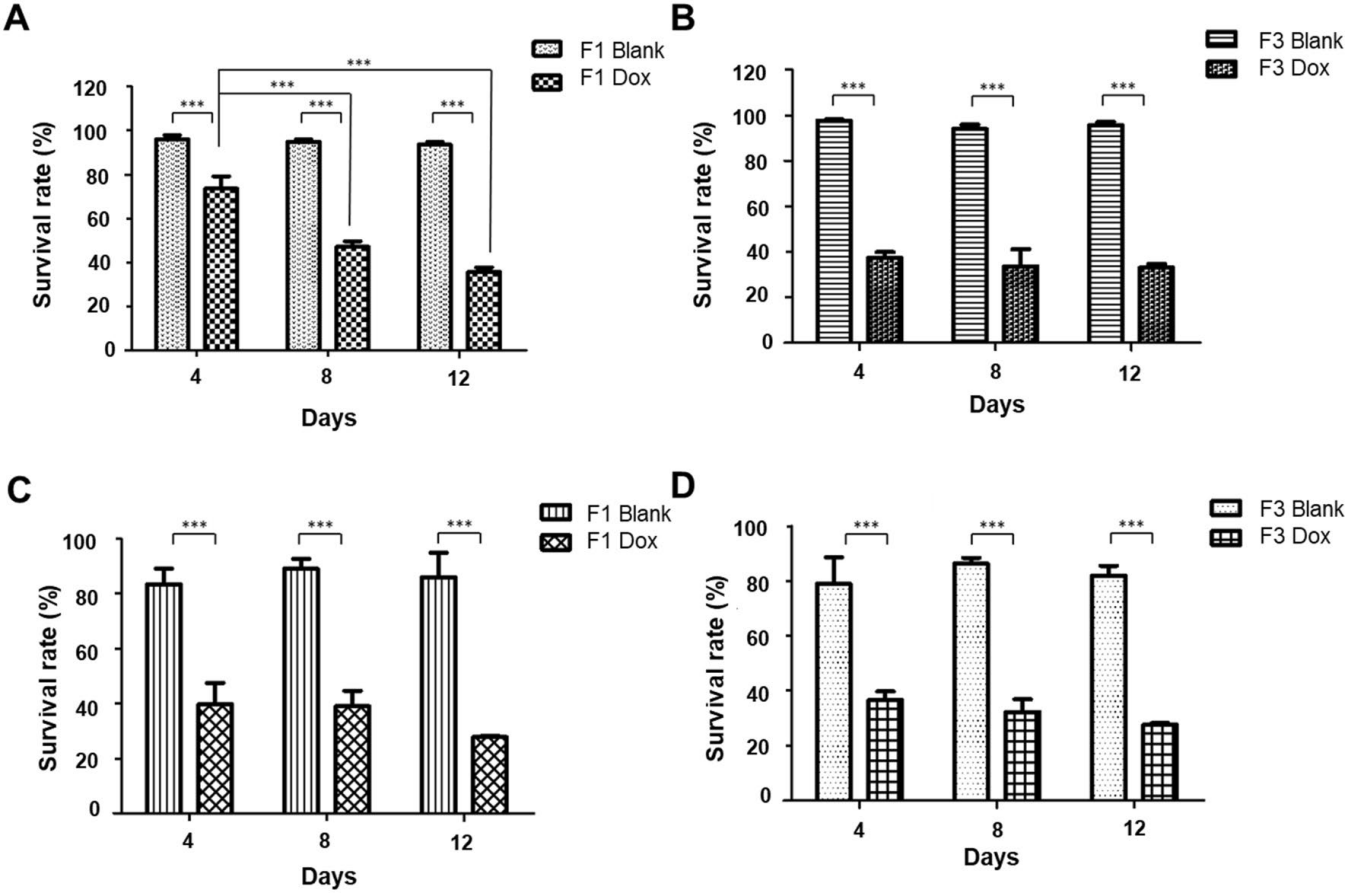
Sustained-release of Dox is profound in F1 compared to F3 calcium alginate microcarriers in Huh-7 and Hep-3B cells. Cells were treated with F1 and F3 calcium alginate microcarriers without (Blank) or with Dox and cell survival rates were measured with trypan blue exclusion method for 4, 8 and 12 days. (**A**,**B**) Huh-7 cells. (**C**,**D**) Hep-3B cells.

In the experimental design, cells were treated with calcium alginate microcarriers without the Dox as a control group to examine whether the calcium alginate microcarriers were toxic to cells. Figure 7A,B show that Huh-7 cells were treated with the F1 and F3 calcium alginate microcarriers without (Blank) or with the Dox (Dox), and cell survival rates on day 4 were 96.00% ± 2.00 and 97.67% ± 0.58, respectively, in the F1 and F3 Blank. The cell survival rate was not affected significantly by the calcium alginate microcarriers without the Dox. Similar results were also observed in another cell line, Hep-3B, as shown in Fig. 7C,D. After treatment with F1 and F3 calcium alginate microcarriers without the Dox for 4 days in Hep-3B cells, the cell survival rates were calculated as 83.33% ± 5.69 and 79.00% ± 9.64, respectively. Although the survival rates were slightly lower than those of Huh-7, it remained nearly up to ~ 80% in Hep-3B cells.

To evaluate the in vitro anticancer activities of the Dox-loaded F1 and F3 calcium alginate microcarriers, the microcarriers were co-cultured with Huh-7 and Hep-3B cells. Figure 7A shows that the cell viability of Huh-7 was significantly decreased over time after treatment with the Dox-loaded F1 microcarriers. The survival rates on days 4, 8, and 12 were 73.67% ± 5.57, 47.00% ± 2.65, and 35.67% ± 2.68, respectively, indicating that the Dox-loaded F1 microcarriers gradually release drug over time and induced cell death. Figure 7B shows that the Dox-loaded F3 microcarriers reduced cell viability, however, the cell survival rates of the drug-loaded microcarrier F3 were very similar on days 4, 8 and 12. These results suggested that on day 4, the F3 Dox-loaded microcarriers rapidly released a concentration of the Dox sufficient to kill more than 50% of cells. After treatment with the F1 Dox-loaded microcarriers in Huh-7 cells, cell survival rates on days 4, 8 and 12 were estimated as 39.67% ± 2.52, 39.00% ± 7.51 and 27.67% ± 1.73, respectively, with a decreasing tendency. Cell survival rates of Hep-3B on days 4, 8 and 12 after treatment with F3 Dox-loaded microcarrier were 36.67% ± 3.055, 32.33% ± 4.618 and 27.67% ± 0.577, respectively, similar to those were observed in the F1 group (Fig. 7C,D). One possible explanation for lower viability in Hep-3B cells after treatment with the Dox-loaded microcarriers may be a longer doubling time of Hep-3B (40 to 50 h) compared to that of Huh-7 cells (~ 24 h), therefore, it may take more time to recover the cell number once Hep-3B cells were killed on day 4. When the number of cells was insufficient, cell division was inhibited by the Dox-loaded microcarriers, resulting in an overall decrease in cell survival rate.

**Figure 8:**
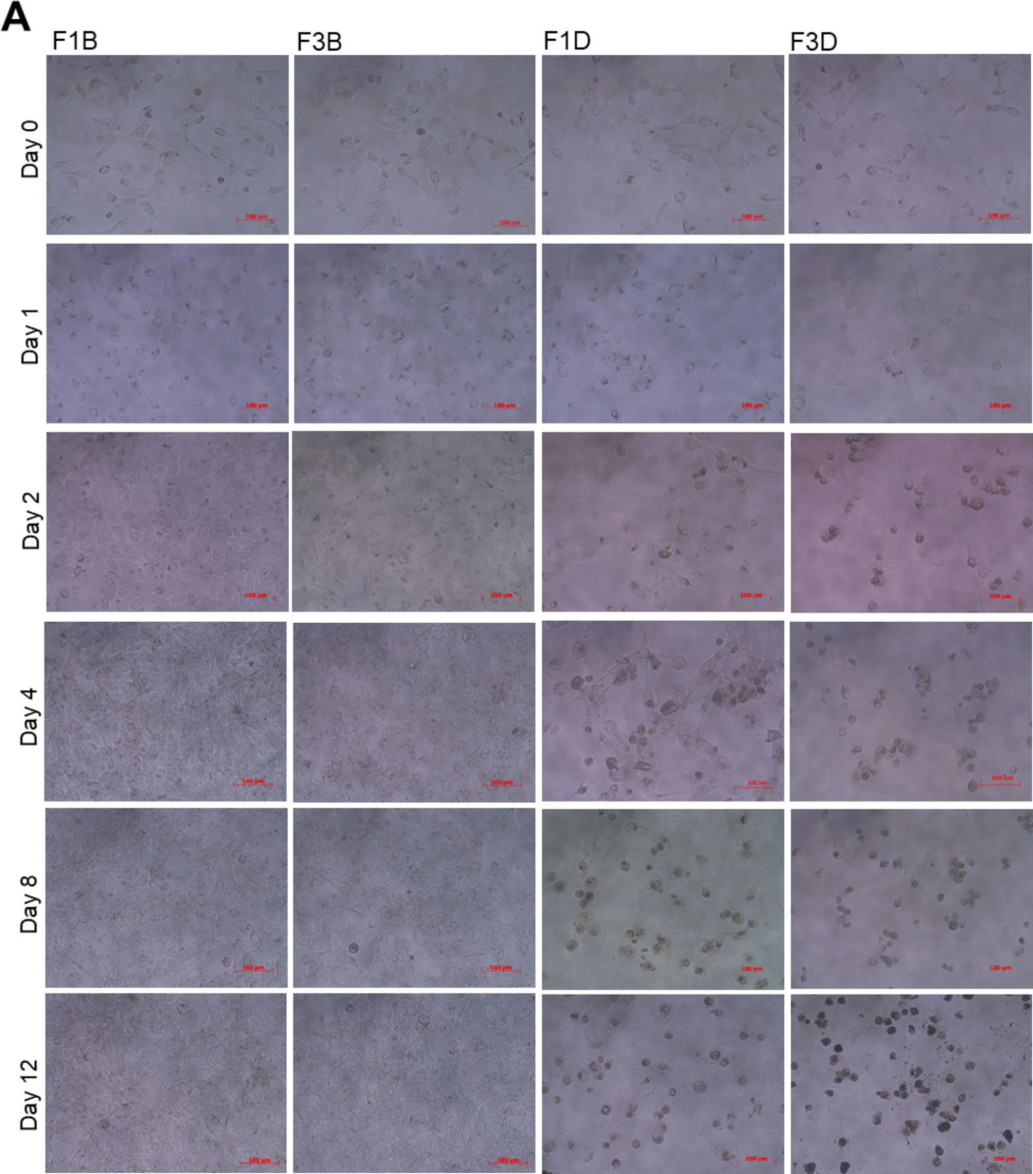

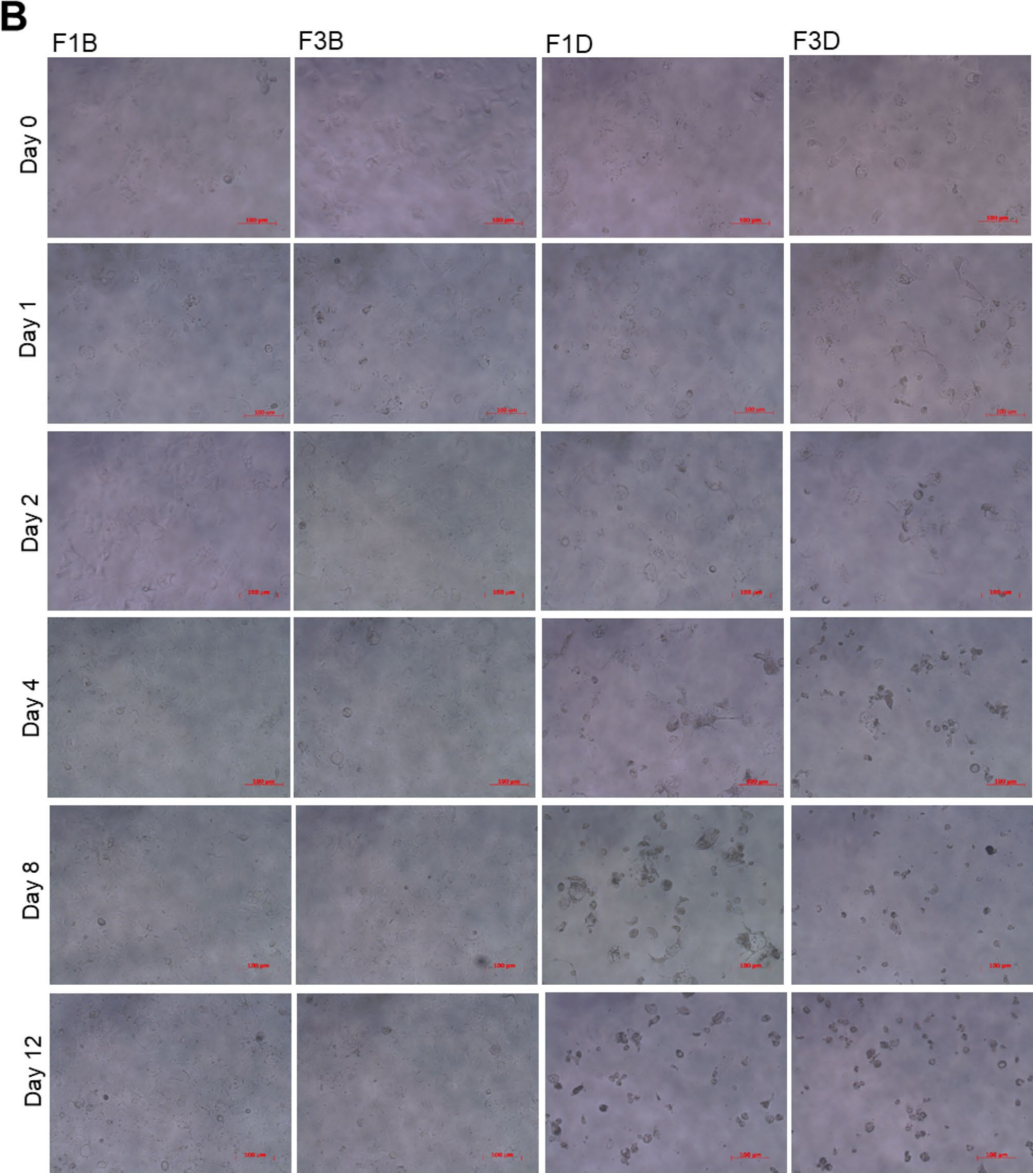
The morphologies of (**A**) Huh-7 cells and (**B**) Hep-3B cells before and after treatments with F1 and F3 Dox-loaded microcarriers at indicated time point. F1B and F3B: before treatment; F1D and F3D: after treatment. On day 4 after treatments with F1 or F3 Dox-loaded microcarriers, even though some cells attached on the culture dishes, most were shrunk into spheres and floated.

In addition, the cell morphologies of both cell lines were not changed after treatments with the F1 (F1B) or F3 (F3B) microcarriers without the Dox for 12 days (Fig. 8A,B). Therefore, calcium alginate microcarriers are non-toxic to cells. Figure 8A (F1D, F3D) and Fig. 8B (F1D, F3D) shows that the morphologies of Huh-7 and Hep-3B cells were not altered on the first day after treatment with the F1 and F3 Dox-loaded microcarriers. On day 4 after treatments with the F1 or F3 Dox-loaded microcarriers, even though some cells attached on the culture dishes, most were shrunk into spheres and floated. On day 12 after treatments with the F1 or F3 Dox-loaded microcarriers, all cells shrunk and died. Thus, treatments with the F1 or F3 Dox-loaded microcarriers for 12 days notably inhibited cell viabilities up to ~ 70% in two distinct HCC-derived cells in vitro. The F1 Dox-loaded microcarriers demonstrated a better sustained-release character to kill cancer cells over time, which is a more ideal drug delivery system.

Taken together, biodegradable calcium alginate microcarriers with a uniform particle size, through dripping-cross-linking and the Taguchi method were successfully prepared. Three important factors including the collection distance, the hardening time and CaCl_2_ solution were identified to be crucial for the quality of products. Signal-to-noise ratio analysis showed that optimal parameters were achieved when cross-linking volume ratio (sodium alginate:CaCl_2_), sodium alginate solution, CaCl_2_ solution, collection distance, flow rate, stirring speed, syringe needle diameter and hardening time were set to 0.1, 2.5 wt%, 6 wt%, 8 cm, 30 mL/h, 150 rpm, 0.25 mm and 2 h, respectively. Our microcarriers were formed by the ionic cross-linking reaction. The calcium alginate microcarriers loaded with the Dox provided a large surface area compared to those without the Dox. Different swelling rates suggested that the calcium alginate microcarriers are pH sensitive. The encapsulation and loaded efficiency of the Dox-loaded calcium alginate microcarriers prepared by the optimal parameters was ~ 40.62% and ~ 3.52%, respectively. Additionally, we provided evidence to show that calcium alginate microcarriers are non-toxic with sustained-release properties. The Dox-loaded calcium alginate microcarriers prepared by optimal parameters eliminate ~ 70% of cancer cells on day 12 after treatments.

## Methods

### Statement

All experiments and methods were performed in accordance with relevant guideline and regulations.

### Chemicals and reagents

Sodium alginate [(C_6_H_7_NaO_6_)_n_, food-grade, MW =  ~ 222 g/mol, macromolecule: 10,000–600,000 g/mol, purity > 90%] and calcium chloride (> 99.99%) were obtained from Sinlong Food’s Additive Inc. (Kaohsiung, Taiwan) and Sigma-Aldrich (St. Louis, MO, USA), respectively. Doxorubicin was purchased from Concord Biotech Limited (Gujarat, India). FBS and Trypsin–EDTA were obtained from Gibco (Carlsbad, CA, USA). Minimum Essential Medium (MEM), Dulbecco's Modified Eagle Medium (DMEM) and antibiotics (10,000 IU/mL penicillin and 10,000 μL/mL streptomycin were attained from HyClone™ Laboratories Inc. (San Angelo, TX, USA). Dimethyl sulfoxide (DMSO) was acquired from Sigma-Aldrich (St. Louis, MO, USA). Sodium alginate solutions were prepared at the concentration of 1.5, 2 and 2.5 wt%, respectively, in deionized water by stirring. The mixture was stirred overnight to completely admix the solution, and the bubbles were fully removed by shaking with an ultrasonic oscillator for 20 min before use. The calcium chloride solution (500 mL) was formulated in deionized water to form 3, 6 and 9 wt% solution, respectively. The calcium chloride solution was sterilized by an autoclave.

### Preparation of microcarriers without or with the Dox

The equipment including the infusion pump, height gage, hot plate, needle and collection region are shown in Supplementary Fig. S3A. The dripping-cross-linking method was used to prepare calcium alginate microcarriers. Supplementary Fig. S3B shows the experimental methods where the sodium alginate solution was placed in a syringe, and the syringe was squeezed by an infusion pump. The sodium alginate solution without or with the Dox (2 mg/mL) was extruded dropwise through a gage nozzle into sterile calcium chloride solution. Mixing of the sodium alginate solution and calcium chloride without or with the Dox induced an ionic cross-linking reaction. In the latter group, the Dox was induced into the microcarriers in the reaction. The microcarriers were stirred at 150 rpm for curing, isolated by filtration, washed twice with sterile water, and kept in 0.06 wt% calcium chloride solution at room temperature.

### Experimental design by the Taguchi method and analysis

Table 1 shows the eight control factors selected in the optimization study for sphere integrity. A standard orthogonal array L_18_ (2^1^ × 3^7^) was designed to investigate this system. L_18_ indicates that the Latin square and the number of experiments, respectively. All studied factors containing two or three levels in all experiments were performed in triplicate. Eight studied variables were investigated to optimize the properties of microcarriers by the Taguchi design and eighteen formulations were prepared according to the orthogonal L_18_ array. Based on previous studies^29^, for sodium alginate and CaCl_2_ concentrations, ranging from 1% to 2.5% and 2% to 10%, resulted in cross-linking volume ration locating at 0.17 and 0.1, respectively. Since different collection distances cause different impact forces for droplets and further affect the sphere shapes, we also considered it as an important parameter. The collection distance which was available to fabricate in spherical or egg shape must be higher than 2 cm^30^, therefore, we designed it as 2, 5 and 8 cm in this study. Due to higher stirring speed, oval or drop-shaped microcarriers could be produced, so 200 rpm was set as our upper limit based on an earlier study^30^. The flow rate and the diameter of the syringe needle must be matched, thus, two parameters were adjusted based on the limits of our equipment. Also, the hardening time was selected as 1, 2 and 3 h for the experiment because the structure of microspheres became stable after 3 h. Our major target was high surface completeness, so the Eqs. (1) and (2) were used to calculate the integrity of the microsphere surface, where ${{\varvec{\eta}}}_{{\varvec{L}}}$, *n*, *yi* and MSD represent *S/N*, experiment repeats, quality of experiment *i* and mean square deviation, respectively.

1 $${\eta }_{L}=-10{\mathit{log}}_{10}\left[\frac{1}{n}\sum_{i=1}^{n}\frac{1}{{y}_{i}^{2}}\right]=-10 {log}_{10}\left(MSD\right)$$

2 $$MSD=\frac{\sum_{i=1}^{n}{({y}_{i}-m)}^{2}}{n}$$

Microcarriers were immersed in PBS for 14 days and the appearance integrity was scored individually, as shown in Table 2, targeting highly intact spheres. After the calcium alginate microcarriers were immersed in PBS for 14 days, they expanded and became larger. Higher scores indicate more intact of spherical surfaces (Supplementary Fig. S4). The combination of the optimal, middle and worst parameters was denoted by the F1, F2 and F3 (Supplementary Table S4). The optimum parameters were determined by Taguchi’s two-step optimization method. The first step was to select the factor/level combination to maximize the response, applied to the experimental data and the *S/N* ratio for each level of the process parameters. The second step was based on the *S/N* ratio and statistical analysis of the results by analysis of variance to assess the importance of the process parameters. The optimized combination of the process parameters has been predicted.

### Characterization of the calcium alginate microcarriers

The calcium alginate microcarriers were designed to be applied as a hydrogel form. To characterize their properties, FTIR was used to obtain infrared spectra of the absorbance of the F1, F2, F3 calcium alginate and sodium alginate microcarriers. The microcarriers were dried at 55 ℃ overnight in an oven and finely grounded with KBr to prepare the pellets under the oil pressure of 15 MPa and spectra were scanned between 4000 and 400 cm^−1^. Furthermore, calcium alginate microcarriers (without or with the Dox) prepared using parameters F1, F2 and F3, respectively, were collected after drying at 55 °C. The microscopic surface features of the calcium alginate were observed using an SEM. Equivalent swelling studies of microcarriers in hydrogels form were performed in pH 7.4 and pH 6.5 PBS at 37 °C. The microcarriers were weighed to 0.04 g (*W*_0_) and placed into a 24-well plate. The calcium alginate microcarriers were separately immersed in PBS with different pH, and positioned in an incubator at 37 °C. After 15 min, the swollen microspheres were immediately weighed (*W*_*t*_) after removal of excess of PBS by using a blotter. This procedure was repeated until the microspheres reached a constant weight. All samples were prepared in triplicate. The swelling rates of the microcarriers were calculated by the following equation:

3 $$Swelling rate\left(\%\right)=\frac{{W}_{t}-{W}_{0}}{{W}_{0}}\times 100\%$$

### Drug encapsulation and loaded efficiencies and in vitro release

The encapsulation efficiency and loaded efficiency of the Dox in the microcarriers were determined spectrophotometrically. About ~ 10 mL of the Dox-loaded sodium alginate solution (2 mg/mL) was placed in a syringe and preparing the Dox-loaded microcarriers. After the calcium alginate microcarriers were prepared, the calcium chloride solution and the Dox-loaded sodium alginate solution remaining in the syringe were collected and calculated. Drug residues in the syringe after dipping and gelation bath were both considered. At wavelength 230 nm shows a strong absorbance of the Dox. Therefore, a calibration curve was generated by a spectrophotometer (NANODROP 2000, ThermoFisher, Waltham, MA, USA). Briefly, the Dox was dissolved in deionized water and 1 μL of each solution was measured at 230 nm. A linear curve was next generated with serial dilutions of the Dox (0.0 to 0.4 mM) and unknown concentrations of the Dox solutions can be calculated (Supplementary Fig. S5). To remove the water and obtain net weight, microcarriers were dried at 55 ℃ overnight in an oven. The encapsulation and loaded efficiencies were calculated by the Eqs. (4) and (5), where *W*_*l*_ is the weight of drug-loaded in the microcarriers (the total drug subtracted residual drug in syringe and gelatin bath), *W*_*td*_ is the total weight of the drug used initially and *W*_*tm*_ is the total weight of the dry microcarriers.

4 $$Encapsulation efficiency\left(\%\right)=\frac{{W}_{l}}{{W}_{td}}\times 100\%$$

5 $$Loaded efficiency\left(\%\right)=\frac{{W}_{l}}{{W}_{tm}}\times 100\%$$

In vitro, drug release was measured in two solutions at 37 °C: PBS and PBS containing 10% FBS. The microcarriers without and with the Dox were prepared as the controls and the experimental groups (F1, F2 and F3), respectively. All were washed three times with deionized water and water was completely removed by suction. Literally, ~ 0.3 g microcarriers were placed onto MILLICELL hanging cell culture inserts (Merck KGaA, Darmstadt, Germany) in 12-well plates. Next, 1.5 mL of PBS or PBS containing 10% FBS were individually added to 12-well plates. Drugs were gradually released from the microcarriers in PBS or PBS containing 10% FBS. At regular intervals, 1 μL of the supernatant from both control and the Dox-loaded groups were sampled and analyzed using a NANODROP 2000 (. Microcarriers without the Dox were used as the calibrator. In order to simulate the in vivo environment, drug release tests were conducted in a 37 °C incubator throughout the experiment. Drug release rate in each group was calculated by Eq. (6), where *Wds* is the total weight of drug in the supernatant.

6 $$Drug release\left(\%\right)=\frac{{W}_{ds}}{{W}_{l}}\times 100\%$$

### In vitro anticancer activity

Two HCC-derived cell lines Huh-7 and Hep-3B were used to evaluate the cell viabilities of the Dox-loaded microcarriers. Cells were maintained in a humidified incubator with 5% CO_2_ at 37 °C with DMEM containing 10% (v/v) FBS and 1% (v/v) antibiotic. Cells (6 × 10^4^) were seeded in a 12-well plate overnight and treated with a microcarrier (without or with the Dox) in a cell culture insert to precisely separate cells from the microcarrier but containing enough median to submerge the microcarrier for drug release, as shown in Supplementary Fig. S6A. Therefore, the gradually released Dox was freely circulated between the upper and lower chambers. At the same time, the wells without cells but with the same microcarrier and medium were simultaneously prepared for the subsequent medium replacement to reach the cumulative Dox concentrations. After 4, 8 and 12 days, cells were collected and subjected to cell viability analysis using trypan blue exclusion assay with an automated cell counter (TC20, Bio-Rad, Hercules, CA, USA).

After cell viabilities were determined on days 4 and 8, the remaining unmeasured well plates were treated as shown in Supplementary Fig. S6B. The microcarrier without the Dox groups were subcultured to 6 × 10^4^ cells/well using the same medium. Media with the cumulative Dox concentrations were replaced every 4 days to reduce experimental errors due to the consumption of nutrients in the medium. One-way analysis of variance (ANOVA) was used to evaluate the significant differences of cell viabilities among different groups, followed by a Scheffe multiple comparison test. A *P* value < 0.05 is considered as statistical significance.

## Supplementary Information

Supplementary Information.

## Data Availability

The data are shown in the main manuscript, supplementary documentation and available to readers.
