# Supplementary material for: Optimization of the spherical integrity for sustained-release alginate microcarriers-encapsulated doxorubicin by the Taguchi method
Source: Sci Rep. 2020 Dec 10;10:21758. doi: 10.1038/s41598-020-78813-1 (PMC7729399; doi:10.1038/s41598-020-78813-1)
Supplement: Supplementary file 1 — Supplementary Information. [file 41598_2020_78813_MOESM1_ESM.pdf]

Optimization of the spherical integrity for sustained-release alginate microcarriers-encapsulated doxorubicin by the Taguchi method

C.T. Pan[1,2,#], S.T. Chien[2,3,#], T.C. Chiang[4], C.K. Yen[1], S.Y. Wang[1], Z.H. Wen[5], C.Y. Yu[6,*], and Y.L. Shiue[4,7,*]

[1]Department of Mechanical and Electro-Mechanical Engineering, National Sun Yat-sen University, Kaohsiung, Taiwan. [2]Institute of Medical Science and Technology, National Sun Yat-sen University, Kaohsiung, Taiwan. [3]Department of Pathology, Kaohsiung Armed Forces General Hospital, Kaohsiung, Taiwan. [4]Institute of Biomedical Sciences, National Sun Yat-sen University, Kaohsiung, Taiwan. [5]Department of Marine Biotechnology and Resources, National Sun Yat-sen University, Kaohsiung, Taiwan. [6]Liver Transplantation Program and Departments of Diagnostic Radiology and Surgery, Kaohsiung Chang Gung Memorial Hospital, and Chang Gung University College of Medicine, Kaohsiung, Taiwan. [7]Institute of Precision Medicine, National Sun Yat-sen University, Kaohsiung, Taiwan

Running title: Dox-encapsulated alginate microcarriers

Keywords: Taguchi method, sodium alginate, microcarrier, doxorubicin, sustained-releasing embolic drug

#These authors contributed equally

*Corresponding to: Yow-Ling Shiue, PhD

Institute of Biomedical Sciences, National Sun Yat-sen University, Kaohsiung 80424, Taiwan; Phone: +886-7-5252000 ext. 5818; Email: shirley@imst.nsysu.edu.tw

*Chun-Yen Yu, MD

Liver Transplantation Program and Departments of Diagnostic Radiology and Surgery, Kaohsiung Chang Gung Memorial Hospital, and Chang Gung University College of Medicine, Kaohsiung 83301, Taiwan; Phone: +886-975056757;

Email: y7192215@ms17.hinet.net

Supplementary documentation

Supplementary Tables

Table S1 Formulations of calcium alginate microcarriers produced by the Taguchi design in a standard orthogonal array L_{18}

Experiment	A	B	C	D	E	F	G	H
**1**	0.17	1.5	3	2	30	100	0.25	1
**2**	0.17	2	3	5	40	150	0.2	2
**3**	0.17	2.5	3	8	50	200	0.15	3
**4**	0.17	1.5	6	2	40	150	0.15	3
**5**	0.17	2	6	5	50	200	0.25	2
**6**	0.17	2.5	6	8	30	100	0.2	1
**7**	0.17	1.5	9	5	30	200	0.2	3
**8**	0.17	2	9	8	40	100	0.15	1
**9**	0.17	2.5	9	2	50	150	0.25	2
**10**	0.1	1.5	3	8	50	150	0.2	1
**11**	0.1	2	3	2	30	200	0.15	2
**12**	0.1	2.5	3	5	40	100	0.25	3
**13**	0.1	1.5	6	5	50	100	0.15	2
**14**	0.1	2	6	8	30	150	0.25	3
**15**	0.1	2.5	6	2	40	200	0.2	1
**16**	0.1	1.5	9	8	40	200	0.25	2
**17**	0.1	2	9	2	50	100	0.2	3
**18**	0.1	2.5	9	5	30	150	0.15	1

A. Cross-linking volume ratio (Sodium alginate: $CaCl_2$), B. Concentration of sodium alginate solution (wt%), C. Concentration of $CaCl_2$ solution (wt%), D. Collection distance (cm), E. Flow rate (mL/h), F. Stirring speed (rmp), G. Syringe needle diameter (mm) and H. Hardening time (h).

Table S2 Appearance score and *S/N* ratio of calcium alginate microcarriers

Experiment	Repetition			Mean	MSD	*S/N*
	1	2	3			
1	2	2	3	2.33	0.204	6.910
2	3	4	4	3.67	0.079	11.040
3	3	3	3	3.00	0.111	9.542
4	3	3	3	3.00	0.111	9.542
5	5	5	5	5.00	0.040	13.979
6	5	5	5	5.00	0.040	13.979
7	4	4	4	4.00	0.063	12.041
8	4	4	4	4.00	0.063	12.041
9	4	4	5	4.33	0.055	12.596
10	4	4	4	4.00	0.063	12.041
11	4	4	4	4.00	0.063	12.041
12	4	4	4	4.00	0.063	12.041
13	4	4	4	4.00	0.063	12.041
14	5	5	5	5.00	0.040	13.979
15	3	3	3	3.00	0.111	9.542
16	4	4	5	4.33	0.055	12.596
17	2	2	2	2.00	0.250	6.021
18	4	4	4	4.00	0.063	12.041

MSD, mean square deviation; *S/N*, signal-to-noise

Table S3 Contribution ratio (ρ%)

**Factor**	**df**	**Sj**	**Vj**	**ρ%**
**A**	1	0.025	0.025	0.038
**B**	2	2.041	1.021	3.086
**C**	2	7.551	3.775	11.416
**D**	2	32.304	16.152	48.839
**E**	2	2.258	1.129	3.414
**F**	2	6.367	3.184	9.627
**G**	2	4.753	2.377	7.186
**H**	2	10.844	5.422	16.395
**Error**	2	-	-	-
**Total**	17	66.144	-	100

A. Cross-linking volume ratio (Sodium alginate: $CaCl_2$), B. Sodium alginate solution concentration, C. $CaCl_2$ solution concentration, D. Collection distance, E. Flow rate, F. Stirring speed, G. Syringe needle diameter and H. Hardening time

Table S4 The combination of the optimal, middle and worst parameters were denoted by the F1, F2 and F3

	**A**	**B**	**C**	**D**	**E**	**F**	**G**	**H**
**F1**	0.1	2.5	6	8	30	150	0.25	2
**F2**	0.1	2	9	5	40	200	0.15	1
**F3**	0.17	1.5	3	2	50	100	0.2	3

A. Cross-linking volume ratio (sodium alginate: $CaCl_2$), **B.** Concentration of sodium alginate solution, **C.** $CaCl_2$ solution concentration, **D.** Collection distance, **E.** Flow rate, **F.** Stirring speed, **G.** Syringe needle diameter, and **H.** Hardening time.

Table S5 Drug encapsulation and loaded efficiencies of the calcium alginate microcarriers

**Formulation**	**Encapsulation efficiency (%)**	**Loaded efficiency (%)**
**F1**	40.617 ± 0.854	3.517 ± 0.129
**F2**	37.605 ± 0.705	3.107 ± 0.135
**F3**	48.047 ± 0.756	4.753 ± 0.245

Supplementary figures

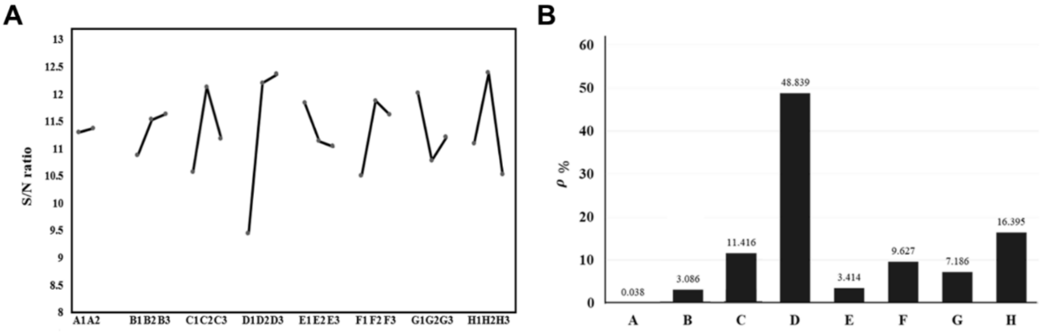

Fig. S1 (A) The factor response graph shows the signal-to-noise (*S/N*) ratio for each parameter. (B) The analysis of the variance was performed based on the *S/N* and expressed as the contribution ratio ρ%. Histogram of contribution ratio (ρ%) indicated that D, H, C and F contributes 48.839%, 16.395% and 11.416%, respectively.

A

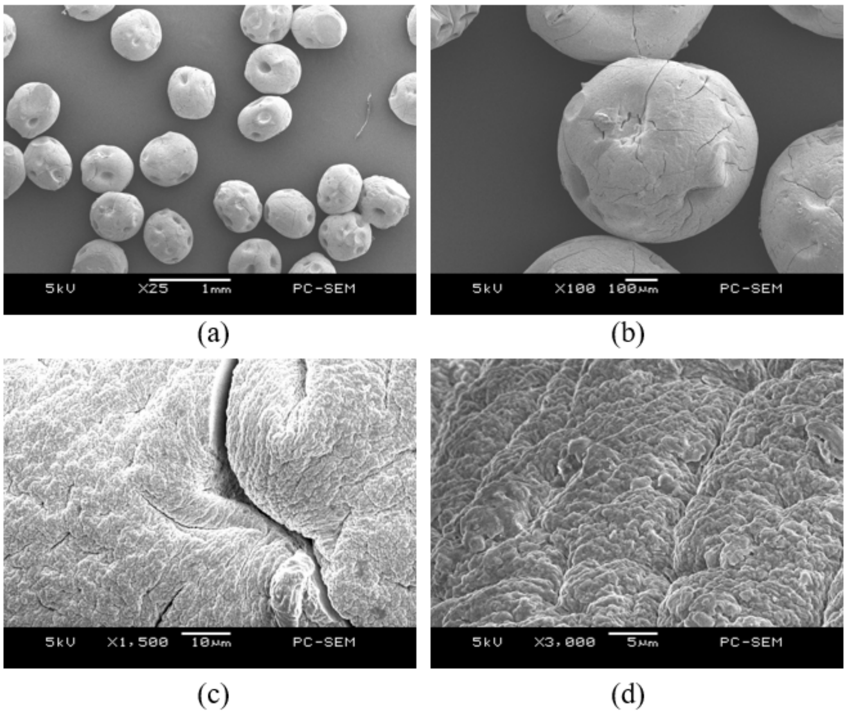
5kV X25 1mm PC-SEM
(a)
5kV X100 100μm PC-SEM
(b)
5kV X1,500 10μm PC-SEM
(c)
5kV X3,000 5μm PC-SEM
(d)

B

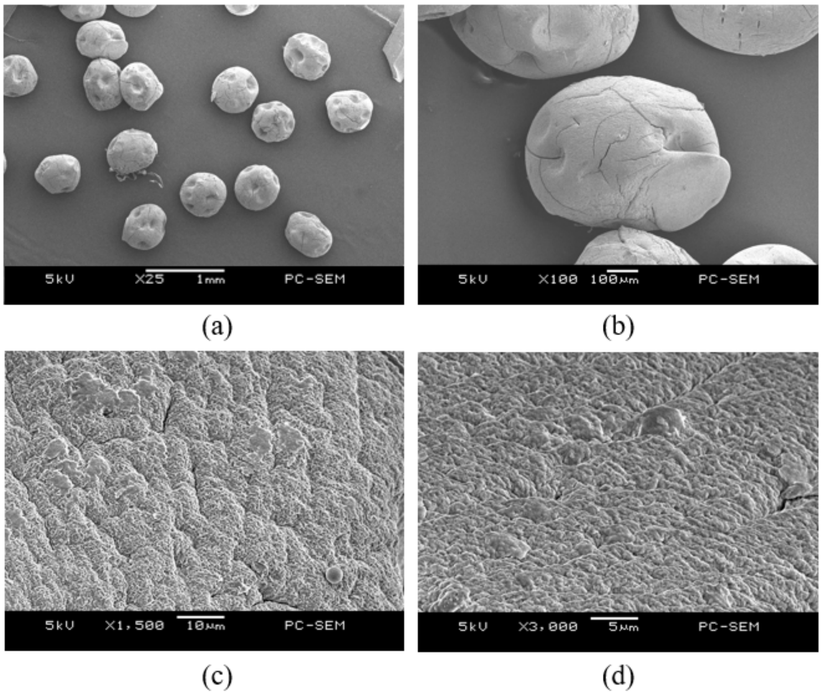
5kV X25 1mm PC-SEM
(a)
5kV X100 100μm PC-SEM
(b)
5kV X1,500 10μm PC-SEM
(c)
5kV X3,000 5μm PC-SEM
(d)

C

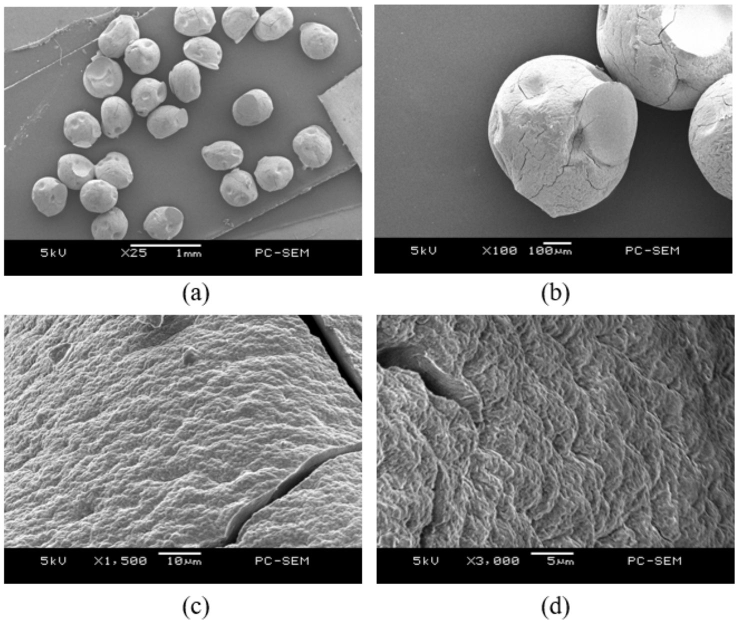

Fig. S2 Scanning election microscope (SEM) images of calcium alginate microcarriers without Dox using parameter of (A) F1, (B) F2 and (C) F3, respectively, and F3 shows dense wrinkles thus increased surface area compared to F1 and F2.

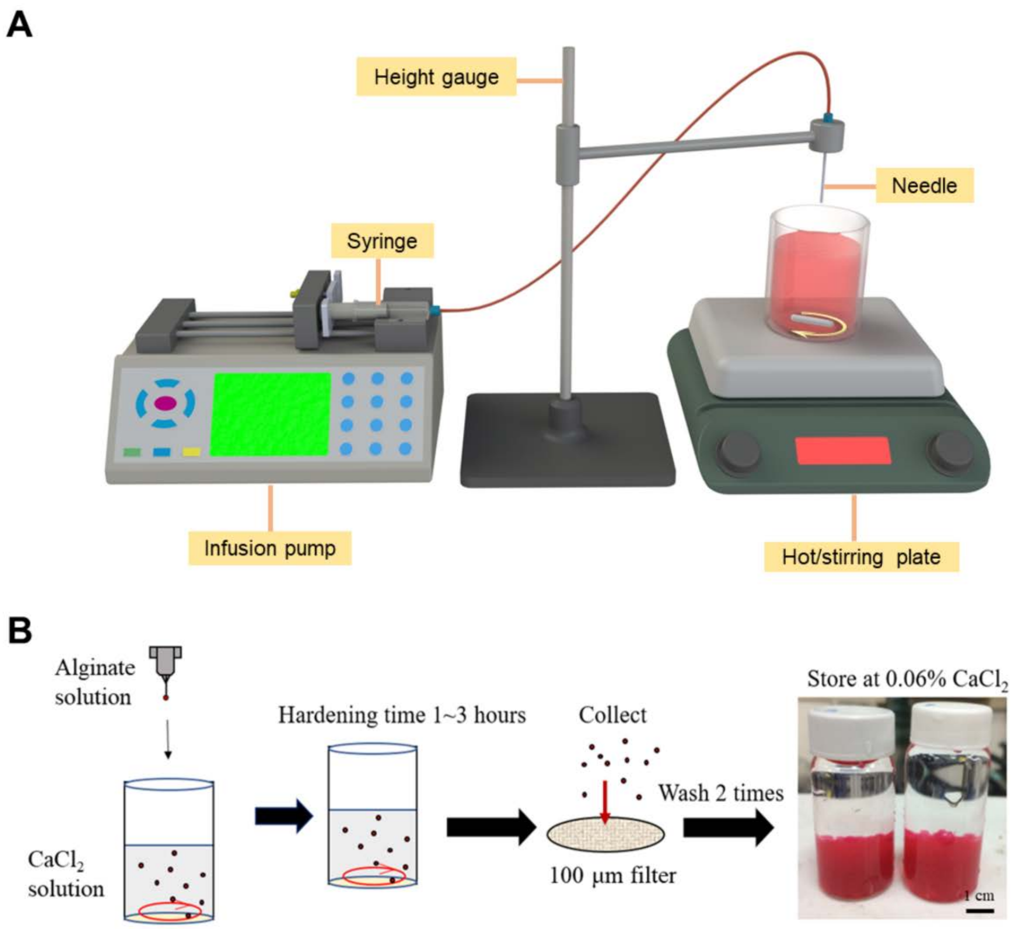

Fig. S3 (A) Schematic diagram of experimental equipment erection. Infusion pump (KDS-100, Kd Science Inc., Holliston, MA, USA) and hot/stirring plate (#07-770-152, ThermoFisher Scientific, Waltham, MA, USA) were used. (B) The dripping-cross-linking method used in this study.

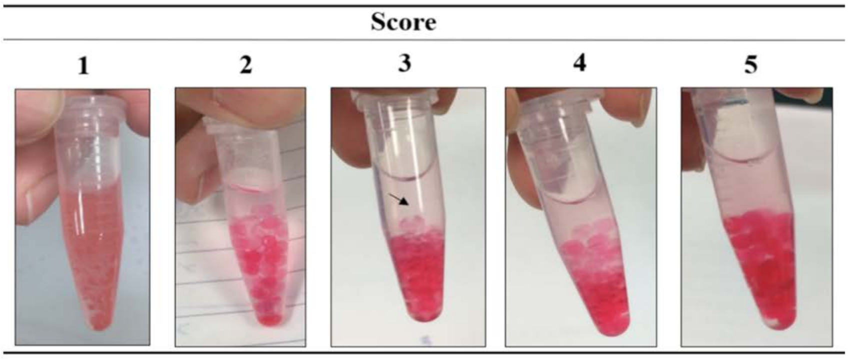

Fig. S4 Higher scores indicate more intact of spherical surfaces, based on the definition in Table 2.

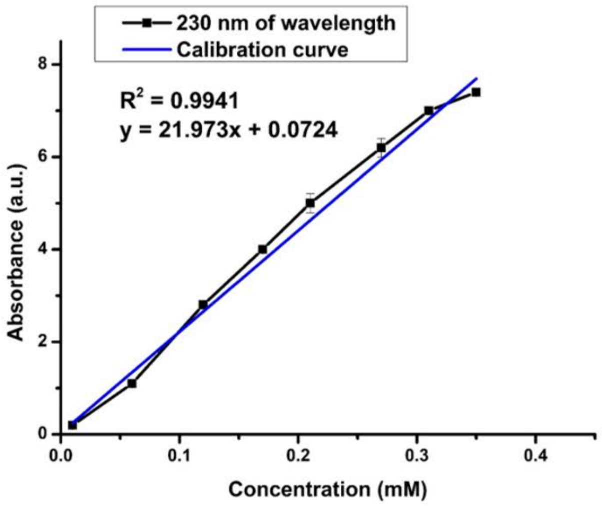

Fig. S5 A calibration curve was generated using different concentration of Dox with a spectrophotometer (NanoDrop™ 2000, ThermoFisher).

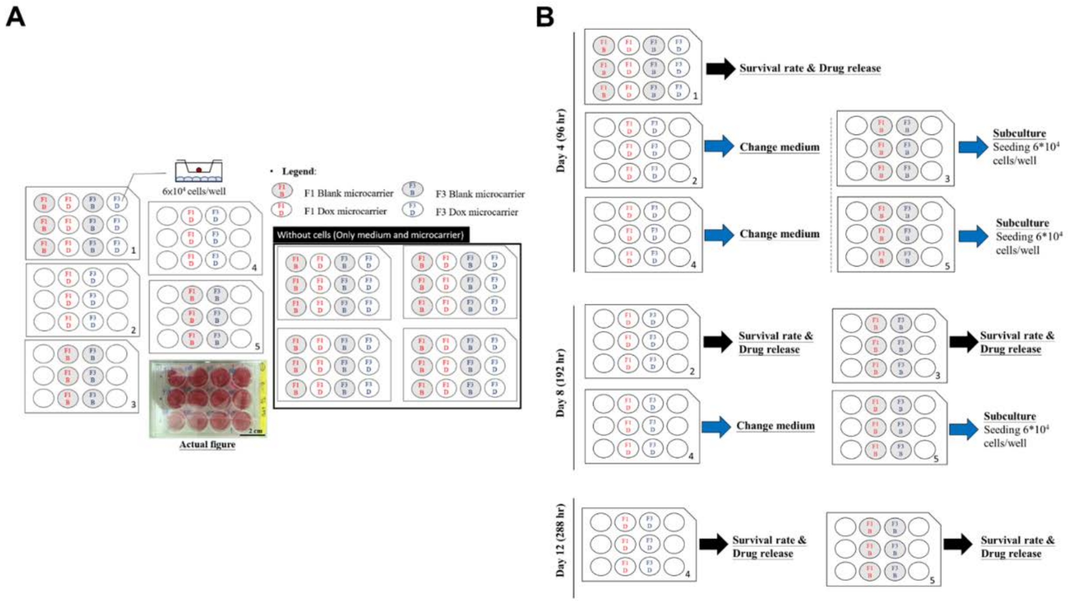

Fig. S6 Schematic diagram of cell configuration of in vitro anticancer activity. Two hepatocellular carcinoma-derived cell lines, Huh-7 and Hep-3B were used to evaluated the cell viabilities of a Dox-loaded microcarrer. (A) Cells (6×10^4) were seeded in 12-well plates overnight and treated with a microcarrier (F1, F2 or F3) without (B: blank) or with Dox (D: Dox) as indicated time point. (B) After co-culture of cells and microcarrier for 4 days, culture medium was replaced with those medium from cell-free Dox-loaded microcarriers and cultured for another 4 days and so on to day 12. The cell viabilities were examined at day 4, day 8 and day 12.
